# Recent advances in *in vitro* fertilization

**DOI:** 10.12688/f1000research.11701.1

**Published:** 2017-08-31

**Authors:** Robert Casper, Jigal Haas, Tzu-Bou Hsieh, Rawad Bassil, Chaula Mehta

**Affiliations:** 1 Division of Reproductive Sciences, Department of Obstetrics and Gynecology, University of Toronto and TRIO Fertility, Toronto, Ontario, Canada; 2 Lunenfeld-Tanenbaum Research Institute, Sinai Health System, Toronto, Ontario, Canada; 3 Department of Physiology, University of Toronto, Toronto, Ontario, Canada

**Keywords:** IVF, Co-enzyme Q10, Letrozole, PGS

## Abstract

The field of assisted reproductive technology is rapidly progressing with many new advances in the last decade. The present review discusses methods to improve oocyte quality in older women and new stimulation protocols that may improve the number of mature oocytes retrieved during an *in vitro* fertilization cycle. We will discuss the present use of pre-implantation genetic screening (PGS) and finally focus on some new methods to determine endometrial receptivity. The focus of this review is to point out areas of technology that may be controversial or are new enough to require proper controlled studies for validation.

## Introduction

In this review, we were asked to update the readers of F1000 on the rapidly advancing field of assisted reproduction. There have been too many new technologies that have been developed over the last 5 years to cover in detail in this review, so we decided to focus on advances that have generated some controversy or that are new enough to require further validation in the future. We plan to discuss methods to improve oocyte quality, especially in older women, together with new stimulation protocols that may improve the number of mature oocytes retrieved during an *in vitro* fertilization (IVF) cycle. We will discuss the present controversy around pre-implantation genetic screening (PGS) and finally focus on some controversial new methods to determine endometrial receptivity. Needless to say, much of what we have to say in this review will reflect our personal opinions in some of these areas.

## Improving oocyte quality: the role of mitochondria

The reproductive capacity of women decreases significantly in the fourth decade, which is directly correlated to an age-related decrease in oocyte quality and quantity ^1^. Fecundity starts decreasing gradually at age 32 and then drops exponentially after 38 ^2^. The fact that live-birth rates from oocyte donation in older women are consistent with the age of the donor suggests that oocyte quality is the major factor responsible for reduced fecundability with aging. The pathways leading to increased loss of ovarian follicles in “old” ovaries are not fully understood, although increased DNA damage due to a less active DNA repair mechanism is a possible trigger for oocyte loss ^3^. The decreased quality of oocytes involves an increased rate of chromosomal aneuploidy with aging predominantly related to meiotic errors during oocyte maturation. The oocyte maturation process involves a combination of nuclear, cytoplasmic, and epigenetic changes, all of which require energy that is provided by the mitochondria via oxidative phosphorylation (OXPHOS) ^4^.

### Co-enzyme Q10 supplementation

The production of ATP via OXPHOS involves a complicated process including 5 complexes located on the inner mitochondrial membrane ^1^. Ubiquinone or coenzyme Q10 (CoQ10) plays an important role in this process, as it has antioxidant properties, controls cellular redox, and affects various signaling pathways ^5, 6^. The concentration of CoQ10 in most tissues decreases after 30 years of age in humans ^7, 8^, and this decline in CoQ10 may contribute to the aging process, since it coincides with the decline in fertility and increased rate of aneuploidies. Ben Meir *et al*. in 2015 showed that supplementation of CoQ10 in an aged animal model delayed decline in ovarian reserve, restored oocyte mitochondrial gene expression, and improved mitochondrial activity with a significant reduction in oocyte aneuploidy. As a result, these aged mice had more oocytes with stimulation, the developmental potential of the oocytes was improved, and more offspring were born compared to old animals receiving placebo ^1^. As a follow-up study, conditional disruption of the gene *PDSS-2*, resulting in isolated CoQ deficiency in the oocytes of young animals, resulted in phenotypic changes characteristic of oocyte mitochondrial dysfunction associated with aging ^1^. By feeding the animals CoQ10, these changes could be reversed.

Since CoQ10 administration in old animals was shown to have beneficial effects on reproductive outcomes, one could speculate that older women might have the same benefits when supplemented with CoQ10 ^1^. At present, there is much research activity in this area. Although the animal model appears promising, the aging process differs greatly between mice and women because of the huge difference in lifespan. CoQ10 administration for 12–16 weeks in mice is likely equivalent to years of use in humans ^1^ and thus additional large-scale clinical studies are needed.

### Mitochondrial transfer

Other attempts to overcome ooplasmic aging have utilized oocyte manipulation at a subcellular level. Cohen *et al*. performed ooplasmic transfer from donor oocytes into mature oocytes of patients who had failed multiple IVF cycles because of poor embryo development and showed healthy live births as a result ^9^. Since cytoplasm contains mitochondria, mitochondria from the donor ooplasm were also transferred into the recipient eggs and were believed to be the most important mediator of improved embryogenesis. Several of the healthy babies tested were found to be heteroplasmic, i.e. to have mitochondrial DNA (mtDNA) derived from the mother and the cytoplasm donor ^10^, and the procedure of ooplasm donation is no longer used. However, autologous mitochondrial transfer has now extended this previous work with ooplasm transfer. In this new technology, mitochondria similar to oocyte mitochondria are isolated from oocyte precursor cells in the superficial epithelial layer of the patient’s ovary and are injected into the patient’s own oocytes at the time of fertilization. This mitochondrial injection has been demonstrated to improve embryo development and lead to live births in women with previous poor embryo development ^11^. This technique, and even the presence of oocyte precursor cells, remains controversial and needs proper randomized control studies for validation.

## Improving oocyte quality with new stimulation and triggering protocols

### Co-treatment with gonadotropins and letrozole in *in vitro* fertilization

Recently, a few studies have demonstrated a potential benefit of the use of the oral agent letrozole together with gonadotropin stimulation in IVF cycles, especially in breast cancer patients going through fertility preservation treatment ^12– 15^. The goal of co-administration of letrozole is to reduce serum estrogen concentrations during ovarian stimulation in breast cancer patients. These studies showed that treatment of breast cancer patients with letrozole and gonadotropins throughout the entire stimulation significantly decreased estradiol concentrations as expected but, interestingly, also increased the number of mature oocytes for cryopreservation compared to controls without breast cancer treated with standard COH ^15^. As far as we know, only breast cancer patients undergoing IVF treatment have been treated with letrozole during the whole stimulation phase so far. In our opinion, however, this protocol is likely an excellent treatment for normal responders undergoing IVF to lower the dose of gonadotropins required to obtain adequate numbers of oocytes for fertilization and to keep estrogen levels closer to the physiologic range.

There are some limited data for the use of letrozole in IVF cycles of normal responders involving co-administration of gonadotropins and letrozole for 5 days in the early follicular phase ^16– 18^. Favorable outcomes related to letrozole were reported, including lower doses of gonadotropin, which decreased the cost of the IVF treatment, and increased numbers of oocytes and mature oocytes while achieving the same pregnancy rate compared to conventional stimulation. More data exist for the use of letrozole in IVF cycles of poor responders. The rationale for co-treatment with letrozole in poor responders is to increase the intrafollicular androgen concentrations, which have been shown to serve as precursors for ovarian estrogen synthesis as well as having a fundamental role in ovarian follicular development by augmentation of FSH receptor expression on granulosa cells ^19^. Co-administration of letrozole and gonadotropins has been described to improve the outcomes in poor responders undergoing IVF cycles ^20– 23^. Garcia-Velasco *et al*. ^24^ in 2005 evaluated the impact of letrozole as an adjuvant treatment in IVF cycles on intraovarian androgens and cycle outcome. They found that adding 2.5 mg of letrozole for the first 5 days of gonadotropin stimulation significantly increased follicular fluid androstenedione and testosterone concentrations and improved IVF cycle outcome. They found a significantly larger number of retrieved oocytes and a significantly higher implantation rate in the letrozole group compared to the control group.

Although the results of these studies are promising, further prospective studies will be needed to confirm the potential benefit of adding letrozole to gonadotropins in both normal and poor responder patients undergoing IVF.

### Pre-treatment with dehydroepiandrosterone/testosterone

Considering the profound effect intraovarian androgens may have on early follicular growth, different protocols have been used to try to increase intrafollicular androgen concentrations in poor responder patients.

Pre-treatment with transdermal testosterone was shown to improve ovarian sensitivity to FSH and follicular response to gonadotrophin treatment in low-responder IVF patients ^25^ and resulted in an increase in the number of cumulus oocyte complexes retrieved as well as improved clinical pregnancy and live-birth rates ^26^.

Gleicher *et al*. ^27^ investigated patients with diminished ovarian reserve who were supplemented with dehydroepiandrosterone (DHEA) for 30–120 days (25 mg 3 times daily). They demonstrated higher AMH levels in the treated patients compared to the non-treated patients, and they also demonstrated improved pregnancy rates. The same group also demonstrated that DHEA may reduce embryo aneuploidy rate ^28^ and also miscarriage rate ^29^. Wiser *et al*. ^30^ performed a prospective randomized controlled study of the effect of DHEA supplementation on IVF outcomes among poor-responder patients. He found an improvement in the embryo quality and a significantly higher live-birth rate in the DHEA group compared with controls. The number of eggs and zygotes was similar in both groups. The use of DHEA for older women or poor responders remains controversial because of the paucity of randomized controlled trials.

## New data regarding a double trigger for *in vitro* fertilization

In most mammalian species, spontaneous ovulation is preceded by a surge of both FSH and LH, which is thought to be necessary for final oocyte maturation and initiation of follicular rupture. At present, standard IVF cycles utilize hCG as a surrogate for the LH surge. In contrast to hCG, the GnRH agonist-induced gonadotropin surge mimics the natural mid-cycle surge and exposes follicles to both LH and FSH ^31, 32^.

Although previous studies suggested that more mature oocytes may be retrieved with GnRH agonist for ovulation triggering compared to hCG ^33^, the GnRH agonist trigger resulted in luteal phase insufficiency caused by lysis of the corpus luteum and poor pregnancy rates ^33– 35^.

Griffin *et al*. ^36^ showed that a combination of hCG and GnRH agonist (double trigger) in women with more than 25% immature oocytes in previous IVF cycles resulted in a significant increase (2 and a half times higher) in the proportion of mature oocytes retrieved. Similarly, Zilberberg *et al*. ^37^ found a significantly higher number of mature oocytes, embryos, and top-quality embryos in a similar group of women using the double trigger instead of hCG alone.

Lin *et al.* ^38^ investigated whether the double trigger could improve the live-birth rate in normal responders undergoing a GnRH-antagonist protocol for IVF compared with hCG alone as the trigger. In their retrospective cohort study of almost 400 cycles, the mean number of MII oocytes retrieved was significantly greater in the dual-trigger group, as was the implantation, clinical pregnancy, and live-birth rates, when compared to the hCG trigger group. All of these studies, albeit retrospective, suggest an advantage to using a GnRH agonist with hCG as a double trigger in IVF cycles, and future prospective studies are needed to confirm.

## Time-lapse imaging

Elective single embryo transfer has been suggested as the most efficient approach to minimize multiple pregnancies resulting from assisted reproduction treatments, and the traditional morphological evaluation has remained the first-line method for selecting the most developmentally competent embryo from an available cohort.

During the last decade, time-lapse imaging (TLI) has emerged as a novel technology that enables continuous evaluation of early embryo development by automated image acquisition every 5–20 minutes and accordingly does not rely on static observations to define a highly dynamic process. Furthermore, it is possible to score embryos without removing them from the incubator, so there is no exposure to changes in light, humidity, temperature, pH, or gas.

Many morphokinetic parameters have been identified to correlate with the embryo's ability to create a pregnancy ^39, 40^, and many different embryo-selection algorithms have been proposed to increase the prediction rate. Recently, Barrie *et al*. ^41^ performed a retrospective observational analysis that demonstrated a need for the development of in-house embryo-selection algorithms that are specific to the patient, treatment, and environment. Their data suggested that currently available algorithms are not clinically applicable and lose their diagnostic value when externally applied.

A recent meta-analysis ^42^ assessed whether TLI resulted in favorable outcomes for embryo incubation and selection compared with conventional methods in clinical IVF. This analysis included 10 randomized controlled studies and concluded that there is insufficient evidence to support TLI as a superior method compared to conventional methods for human embryo incubation and selection. A well-designed RCT is still needed to evaluate the effectiveness of the clinical use of TLI.

## Pre-implantation genetic screening for aneuploidy

The objectives of PGS for aneuploidy are to select embryos with the highest chance of implantation, to facilitate elective single embryo transfer, and to reduce the risk of chromosomal abnormalities in the offspring. The current embryo biopsy technique for PGS, day 5 multi-cell trophectoderm biopsy, has replaced the old day-3 single blastomere biopsy and is thought to have a much-improved embryo implantation rate. The genetic testing methods for PGS have also achieved higher accuracy with the progression of test platforms from FISH, to aCGH, SNP arrays, Q-PCR, and the current next-generation sequencing (NGS) technology ^43^. PGS may demonstrate abnormal results in more than three-quarters of embryos tested. However, recent research showed that some of these diagnosed abnormal embryos may have the potential to develop into healthy babies ^44^. Embryo mosaicism and the trophectoderm sampling technique presently utilized are two major reasons for the possible inaccuracy of PGS.

Embryo mosaicism is an extremely controversial topic at the moment. Mosaicism of the chromosomal complement in an embryo can be observed at different stages of embryonic development and is thought to arise during mitotic cell divisions after fertilization. Mosaic cells might reside within the inner cell mass, in the trophectoderm, or in both. Also, the distribution of mosaic cells in the blastocyst can be local, patchy, or uniform ^45, 46^. Animal studies found a wide range of mosaic embryos. A recent study using discarded human embryos found similar wide ranges of embryo mosaicism, ranging from 20 to 90% of embryos ^47^. Until now, there is not enough clinical data to predict the fate of the embryos with mosaicism, and there is a clinical and ethical debate around whether embryos determined to be chromosomally mosaic could, or should, be transferred.

Animal studies have shown that some mosaic embryos can implant and develop into healthy babies through self-correction. A few clinical reports so far have demonstrated normal pregnancies after transferring abnormal PGS embryos. Lledo *et al*. used aCGH to re-evaluate trophectoderm biopsy samples and found that 13.4% of embryos previously diagnosed as euploid were mosaic ^48^. The clinical pregnancy rate was thought to be similar between mosaic and euploid embryo transfers, but the miscarriage rate may be higher in the mosaics. Fragouli, using NGS to analyze trophectoderm biopsies, found a lower pregnancy rate in mosaic embryos compared to euploids ^44^. Both studies, however, provided evidence that embryos with certain degrees of mosaicism may develop into healthy babies.

Another controversy concerns the accuracy of PGS as it is now practiced. A recent computer modeling study showed that a single trophectoderm biopsy of 5 to 6 cells cannot accurately estimate the degree of embryo mosaicism, casting into doubt the entire value of PGS ^49^. Possible ways to solve this problem include multiple trophectoderm biopsies or, better yet, biopsy of the inner cell mass ^50^. However, the safety of removing cells from the inner cell mass is not yet established.

Assuming an accurate method of detecting mosaic embryos, an ethical controversy is whether the transfer of these embryos would carry a high risk of miscarriage. On the other hand, discarding aneuploid and mosaic embryos using our present biopsy techniques could potentially result in the loss of embryos that have the potential to develop into a normal baby ^51^. This area of controversy will not be solved in the near future.

### mtDNA content in blastocysts

An offshoot of PGS and trophectoderm biopsy is the ability to determine the mean mtDNA copy number in the blastocyst. Elevated mtDNA copies at the blastocyst stage have been found to be associated with poorer clinical outcome ^52, 53^. Euploid embryos with relatively low levels of mtDNA at the blastocyst stage were observed to have a higher implantation rate compared to blastocysts with a relatively higher copy number of mtDNA. In keeping with the latter observation, blastocysts from younger patients have been found to have lower average copy numbers of mtDNA compared to older patients ^52, 54^. This difference was evident when all blastocysts were considered together but also when chromosomally normal and abnormal embryos were considered separately. This finding raises the question of whether mitochondria might play a direct role in the decline of female fertility with age. Fragouli *et al*. ^54^ examined mtDNA quantity in relation to chromosome status. They found that chromosomally abnormal blastocysts tended to contain significantly larger amounts of mtDNA compared to those which were characterized as being euploid. The elevated mtDNA levels in the abnormal and the older embryos might be a consequence of a compensatory mechanism aimed to increase the ATP generation of compromised mitochondria of reduced function. At present, the analysis of mtDNA copy number at the blastocyst stage as a way to improve live-birth rates is controversial, and further research is ongoing.

## New approaches to assess the receptivity of the endometrium

Embryo implantation occurs following complex synchronized physiological and biochemical interactions between the blastocyst and the endometrium ^55^. This occurs only if the endometrium is in a receptive state. In humans, an individually defined period called the “window of implantation” (WOI) spans 2–4 days during the mid-luteal phase ^56^. Two recent methods used in IVF clinics for the assessment of endometrial receptivity are ultrasound measurement of subendometrial wave frequency and a microarray analysis of putative implantation-associated gene expression, the so-called endometrial receptivity array (ERA).

### Ultrasound assessment of endometrial receptivity

Early studies using transvaginal ultrasound (TVUS) measurement of endometrial thickness hoped to replace invasive techniques like endometrial biopsy for the assessment of endometrial receptivity. A pre-ovulatory endometrium of 7 mm is considered as the cut-off thickness below which suboptimal implantation occurs ^57– 59^. However, pregnancies have been reported in patients with an endometrial thickness as low as 4 mm ^60^. A meta-analysis in 2014 concluded that endometrial thickness by itself did not have a high enough positive predictive value for pregnancy ^61^.

More recent work has focused on TVUS measurement of the subendometrial contractility, observed as “endometrial waves”, that are hormonally responsive in their patterns and propagation directions ^62– 64^. In the follicular phase, these peristaltic waves are directed from the fundus to the cervix (FC waves), but in the late follicular and the ovulatory phase these waves are directed from the cervix to the fundus (CF waves) to assist sperm transportation to the fallopian tube. Following ovulation is a phase of uterine quiescence which would support embryo implantation ^65^. During assisted reproductive technology procedures, supraphysiological estradiol levels are generally reflected by increased wave activity. Fanchin *et al*. observed an inverse correlation between the frequency of subendometrial waves in the luteal phase and pregnancy outcomes ^66^. Increasing progesterone exposure was shown to result in diminished wave activity and improved pregnancy rates. Despite these interesting observations, most IVF units at present do not use ultrasound to assess endometrial wave activity. However, in our opinion, this non-invasive tool could be especially valuable in patients with recurrent implantation failure or with inflammatory conditions such as endometriosis to ensure uterine quiescence prior to the embryo transfer.

### Molecular test of endometrial receptivity

Endometrial histological dating described by Noyes *et al*. in 1950 ^67^ was considered the gold standard for determining the WOI, thought to be around day 20 to 22 of an idealized 28-day cycle. Endometrial deficiency or “out-of-phase” endometrium is thought to occur in as many as 1 in 4 patients ^68^. Multiple randomized studies have cast doubt on the reproducibility of the analysis of endometrial biopsy samples by histologic dating ^69– 71^. A new technique has developed from the transition to microarray molecular analysis using a customized array to identify markers of endometrial receptivity. The ERA based on the analysis of the expression of 238 genes thought to be involved in endometrial implantation may lead to the determination of the personalized WOI ^72^. This test is done by obtaining endometrial biopsy samples on day LH surge + 7 in a natural cycle or the 6 ^th^ day of progesterone administration during an HRT cycle. Results are expressed as pre-receptive, receptive, or post-receptive. If the result is non-receptive, which happens in 1 in 4 patients ^73^, the embryo replacement timing is adjusted, enabling personalized embryo transfer ^74^. This technique, although offered commercially, still requires large-scale randomized studies for validation and the results remain controversial.
